# SPCS: a spatial and pattern combined smoothing method for spatial transcriptomic expression

**DOI:** 10.1093/bib/bbac116

**Published:** 2022-04-04

**Authors:** Yusong Liu, Tongxin Wang, Ben Duggan, Michael Sharpnack, Kun Huang, Jie Zhang, Xiufen Ye, Travis S Johnson

**Affiliations:** 1 College of Intelligent Systems Science and Engineering, Harbin Engineering University, Harbin, Heilongjiang 150001, China; 2 Department of Computer Science, Indiana University Bloomington, Bloomington, IN 47408, USA; 3 Department of Medicine, Indiana University School of Medicine, Indianapolis, IN 46202, USA; 4 Department of Pathology, University of California San Francisco, San Francisco, CA 94143, USA; 5 Department of Biostatistics and Health Data Science, Indiana University School of Medicine, Indianapolis, IN 46202, USA; 6 Regenstrief Institute, Indianapolis, IN 46202, USA; 7 Department of Medical and Molecular Genetics, Indiana University School of Medicine, Indianapolis, IN 46202, USA; 8 Indiana Biosciences Research Institute, Indianapolis, IN 46202, USA

**Keywords:** spatial transcriptomics, imputation, two-factor expression smoothing, *k*-nearest neighbors, tissue region partition, pancreatic ductal adenocarcinoma, dorsolateral prefrontal cortex, high-grade serous ovarian cancer

## Abstract

High-dimensional, localized ribonucleic acid (RNA) sequencing is now possible owing to recent developments in spatial transcriptomics (ST). ST is based on highly multiplexed sequence analysis and uses barcodes to match the sequenced reads to their respective tissue locations. ST expression data suffer from high noise and dropout events; however, smoothing techniques have the promise to improve the data interpretability prior to performing downstream analyses. Single-cell RNA sequencing (scRNA-seq) data similarly suffer from these limitations, and smoothing methods developed for scRNA-seq can only utilize associations in transcriptome space (also known as one-factor smoothing methods). Since they do not account for spatial relationships, these one-factor smoothing methods cannot take full advantage of ST data. In this study, we present a novel two-factor smoothing technique, spatial and pattern combined smoothing (SPCS), that employs the *k*-nearest neighbor (kNN) technique to utilize information from transcriptome and spatial relationships. By performing SPCS on multiple ST slides from pancreatic ductal adenocarcinoma (PDAC), dorsolateral prefrontal cortex (DLPFC) and simulated high-grade serous ovarian cancer (HGSOC) datasets, smoothed ST slides have better separability, partition accuracy and biological interpretability than the ones smoothed by preexisting one-factor methods. Source code of SPCS is provided in Github (https://github.com/Usos/SPCS).

## Introduction

Mammalian tissue is highly heterogeneous with phenotypes that depend on their spatial distribution [1, 2]. Until recently, studies of tissue heterogeneity have either sacrificed spatial relationships (e.g. scRNA-seq) or produced low-dimensional measurements [e.g. immunohistochemistry (IHC)] [3–7]. Novel ST techniques allow whole transcriptome profiles to be measured while preserving spatial relationships [8, 9]. These techniques have already been profoundly useful in understanding tumor [10–12] and non-tumor tissue [13–16] heterogeneity. However, improvements in ST library preparation [17], sequencing techniques and bioinformatic analysis pipelines [18] are still necessary and ongoing in contrast to more established scRNA-seq standard practice protocols [19, 20].

The most widely utilized ST technologies are based on highly multiplexed sequence barcoding, which suffers from expression noise and dropout events [21, 22]. Barcoding-based scRNA-seq data suffer from similar limitations, while plate- and *in vitro* transcription-based techniques, such as Smart-seq2 [23] and CEL-seq2 [24], respectively, provide more representative expression profiles per cell at the cost of fewer cells measured per experiment. As a result, a multitude of techniques have been developed to impute the missing expression values and smooth the noise that comes directly from the barcode-based non-spatial scRNA-seq. SAVER [25] and MAGIC [26] use sets of correlated genes and relative cell similarity in transcriptome space to impute the dropout events and eliminate other types of expression errors via machine learning techniques. These methods are termed as ‘one-factor methods’, given that they only incorporate expression values. The smoothened expression values give more accurate representations of the true underlying RNA abundances than the raw read counts. ST data have the advantage of providing spatial relationships that can be used in addition to transcriptomic similarity for smoothing based on the assumption that nearby cells will have more similar expression profiles than distant cells.

Here, we present a novel two-factor smoothing method, termed spatial and pattern combined smoothing, i.e. SPCS, specifically designed for ST data, which utilizes both the associations of spatial locations in transcriptome space (expression pattern knowledge) and in Euclidean space (spatial knowledge). By performing SPCS on multiple ST slides from PDAC, DLPFC and HGSOC datasets, smoothed ST slides have better separability, partition accuracy and biological interpretability than the ones smoothed by preexisting one-factor methods.

## Methods

### Datasets

The datasets that we use in this study include two real-world ST datasets, PDAC [10] and DLPFC [14], and a simulating dataset generated from HGSOC single-cell datasets [27]. For the two real-world datasets, PDAC includes 10 ST slides sourced from the traditional ST platform, while DLPFC is a Visium platform dataset with 12 slides. All the data in these datasets consist of two different matrices containing gene expressions and spatial coordinates. One matrix consists of the gene expression values for each spatial barcode hybridized from its corresponding spot on the ST slide. The other matrix contains the spatial locations in 2D space for each spot’s spatial barcode. Using these two matrices, we can generate a 2D representation of each gene’s expression value throughout the biopsied tissue section. Because these datasets are sourced from different ST platforms, i.e. traditional ST platform and new developed Visium platform, we can explore the influence of smoothing methods more comprehensively. A detailed statistical summary of the data we used is provided in Supplementary Table S1 available online at https://academic.oup.com/bib.

To better explore the ability of smoothing methods to deal with outlier spots, we designed a simulation experiment based on those used in the BayesSpace study [28]. Simulated ST data are based on HGSOC single-cell datasets and an immunofluorescence stained image of an ovarian cancer biopsy. In the original single-cell analysis of the HGSOC dataset, all the cells were divided into 15 clusters by the DBSCAN clustering method and annotated [27]. Considering the limited number of cells, we only used some of the slides. Ground-truth cluster labels were derived from single-cell level annotation of tumor and stroma compartments within the image. To make the simulated data reflect biology, we separated the slide into four clusters: intratumor (including dendritic and fibroblast cells), stroma (corresponding to macrophage cells) and two tumor clusters (associated with two different malignant cell clusters). Detailed information about ground-truth clusters is provided in Supplementary Table S2 available online at https://academic.oup.com/bib. To test the ability of different smoothing methods to find outlier spots, we randomly mixed 5% of other cell types as perturbation for each spatial cluster. We generated 10 sets of simulating data in the simulation analysis.

### SPCS of spatial transcriptomic expression

**Figure 1:**
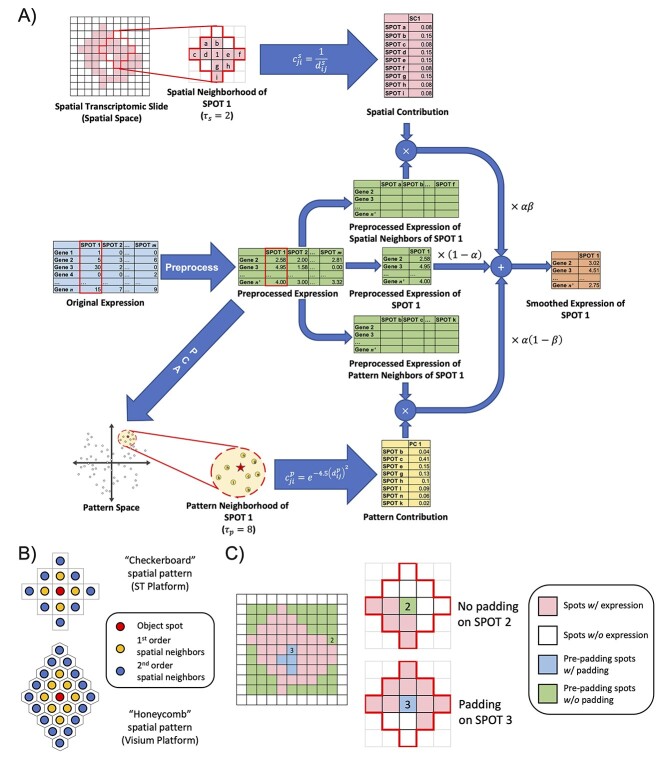
Workflow of our proposed SPCS smoothing method. (**A**) An example of smoothing. In this sample, SPOT 1 is the object spot that is going to be smoothed. For ST slides, preprocessing steps normalize the expressions and filter out genes with zero expression in most spots. Spatial neighborhood is second-order neighborhood of SPOT 1 with nine spots. Spots, where all gene expression is 0, are treated as non-tissue regions and are excluded from spatial neighborhood. Pattern neighborhood consists of the top eight spots with the most similar expression pattern to SPOT1. Both spatial and pattern contributions are normalized, which means the total contribution of all neighbors is =1. Smoothing is performed by integrating parameter- and contribution-weighted expression of the object spot itself and both its spatial and pattern neighbors. (**B**) Shape of second-order spatial neighborhood for ST and Visium platforms. Spatial neighborhood is determined by Manhattan distance. Spots with the same Manhattan distance to object spot belong to the same order of spatial neighborhood. Due to the different geometries of spots, the shape of spatial neighborhood could be different in different platforms. (**C**) Padding strategy of SPCS. A missing spot will not be padded unless there are >50% of non-missing spots inside its spatial neighborhood.

For each spot on an ST slide, there exists not only the gene expression but also its spatial positions. This means we can improve the quality of the expression values within each specific spot using the relative similarity to the other spots based on both expression pattern and spatial location on the ST slide. To achieve this goal, we propose a kNN-based method, SPCS, to perform smoothing and padding. We display the procedure of the SPCS method in Figure 1.

In our method, we obtained the smoothed expression of each spot by integrating the contribution-weighted expression of its pattern and spatial neighbors. Let $\mathbf{X}_i$ be a vector of gene expression values for spot $i$, smoothed expression $\mathbf{X}_i^{\prime }$ can be calculated by the following:

(1) \begin{equation*} \mathbf{X}_i^{\prime }=\left(1-\alpha \right)\mathbf{X}_i+\alpha \left(\beta \frac{\sum_{j\in \mathbf{N}_\mathrm{s}(i)}{c}_{ji}^s{\mathbf{X}}_j}{\sum_{k\in \mathbf{N}_\mathrm{s}(i)}{c}_{ki}^s}+\left(1-\beta \right)\frac{\sum_{j\in \mathbf{N}_\mathrm{p}(i)}{c}_{ji}^\mathrm{p} \mathbf{X}_j}{\sum_{k\in \mathbf{N}_\mathrm{p}(i)}{c}_{ki}^\mathrm{p}}\right). \end{equation*}

In Equation (1), there are two ratio parameters $\alpha$ and $\beta$. $\alpha$ is used to regularize the ratio of original expression to the corrected expression, which avoids the expression of the object spot becoming over-smoothed. $\beta$ is used to adjust the ratio of spatial-based to pattern-based smoothing for different applications. $\mathbf{N}_\mathrm{s}(i)$ and $\mathbf{N}_\mathrm{p}(i)$ are the spatial and pattern neighborhoods of the object spot $i$, respectively. The size of the neighborhoods can be determined by parameters ${\tau}_\mathrm{s}$ and ${\tau}_\mathrm{p}$, which also should be specified in advance. ${c}_{ji}^\mathrm{s}$ and ${c}_{ji}^\mathrm{p}$ represent the spatial and pattern contributions of a corresponding neighbor spot $j$ to the object spot $i$. For traditional ST platform-based PDAC dataset, we set $\alpha =0.6,\beta =0.4,{\tau}_\mathrm{s}=2$ and ${\tau}_\mathrm{p}=16$; and for the Visium platform-based DLPFC and simulating datasets, we make ${\tau}_\mathrm{s}=4$ due to the larger number of spots. We will display the influence of these parameters on data separability and discuss the selection of them in the Discussion section. In addition, SPCS will also fill the missing spots using multiple non-missing spatial neighbors. The source code of our proposed SPCS method is provided in our Github repository (https://github.com/Usos/SPCS). In the next section, we introduce the detailed mathematical definitions of neighborhood, neighborhood contribution and our missing spot padding strategy.

### Pattern neighborhood

ST data are a type of transcriptomic data that measure gene expression patterns similar to scRNA-seq. Like some scRNA-seq data, where cells can be localized to tissue location of origin, spots that have a similar expression pattern are more likely to belong to the same region in a tissue. Therefore, smoothing the expression of a given spot using other spots with a similar gene expression can improve data quality and is similar to one-factor smoothing methods [25, 26] designed for scRNA-seq. A group of the most similar spots based on the expression ‘pattern’ of the spot can be defined as that spot’s ‘pattern neighborhood’. Here, we provide the explicit definition of the pattern neighborhood used by the SPCS method.

**Definition 1** (Pattern neighborhood): $\mathbf{S}_\mathrm{p}$ is the gene expression pattern space of an ST slide; $i,j,k\in \mathbf{S}_\mathrm{p}$ are different spots; ${d}_{ij}^\mathrm{p}$ and ${d}_{ik}^\mathrm{p}$ are pattern distances between spots $i,j$ and $i,k$, respectively. $\mathbf{N}_\mathrm{p}(i)$ is the ${\tau}_p$ pattern neighborhood of spot $i$ if $\mid \mathbf{N}_\mathrm{p}(i)\mid ={\tau}_\mathrm{p},\forall j\in \mathbf{N}_\mathrm{p}(i),\forall k\in \mathbf{S}_\mathrm{p}-(\mathbf{N}_\mathrm{p}(i)\, \cup \{i\})$ s.t. ${d}_{ij}^\mathrm{p}<{d}_{ik}^\mathrm{p}$.

For gene expression data, the overall shapes of gene expression patterns are of greater interest than the individual magnitudes of each feature [29]. Hence, we used the Pearson correlation distance to measure the pattern distance between different spots. Let ${\rho}_{ij}$ represents Pearson correlation coefficient (PCC) of coordinate of spots $i$ and $j$ in pattern space, Pearson correlation distance ${d}_{ij}$ of spots $i$ and $j$ is given as follows [30]:

(2) \begin{equation*} {\displaystyle \begin{array}{c}{d}_{ij}= \mathrm{1}-{\rho}_{ij}.\end{array}} \end{equation*}

In ST data, some genes are expressed at identical or near-identical levels that lack the variance to establish an accurate pattern neighborhood. Therefore, we used principal component analysis (PCA) [31] to transform the expression of spots into a 10D principal component space before smoothing. These uncorrelated components with the largest variance from our PCA are considered as the gene expression pattern space.

### Spatial neighborhood

In contrast to scRNA-seq data, ST data provide the spatial position for each spot in the slide. Regions in proximity on histopathology slides are more likely to be the same tissue type. Aside from the pattern associations between spots, we can also use spatial associations to smooth the expression as a second factor. We define the group of spots that are spatially near a given spot as the ‘spatial neighborhood’ of that spot, which is defined explicitly below.

**Definition 2** (Spatial neighborhood): $\mathbf S$ represents the set of spatial location indices of an ST slide, and $i,j\in \mathbf S$, ${d}_{ij}^\mathrm{s}$ is the spatial distance between spots $i$ and $j$. $\mathbf{N}_\mathrm{s}(i)$ is the ${\tau}_\mathrm{s}$ spatial neighborhood of spot $i$, if $\forall j\in \mathbf{N}_\mathrm{s}(i)$ s.t. ${d}_{ij}^\mathrm{s}\le{\tau}_\mathrm{s}$.

ST spots are spatially distributed in a checkerboard or honeycomb pattern. Due to the geometric patterns inherent to ST spot layout, Manhattan distance is a suitable metric to measure the spatial distance between spots inside an ST slide. Thus, we chose Manhattan distance as the spatial distance to define our spatial neighborhood. Due to the difference in the spatial pattern of spots, the shape of spatial neighborhood could be different in different platforms. Figure 1**B** illustrates the shape of second-order neighborhood of both traditional ST platform (checkerboard spatial pattern) and Visium platform (honeycomb spatial pattern).

### Contribution of neighbors on smoothing

Different neighbors in the spatial or pattern neighborhood will have different impacts on the smoothing for a given spot, which we refer to as ‘contribution’. Since the definitions of spatial and pattern distance are different, we model the corresponding contributions in different ways. The contributions of both spatial and pattern neighbors are still comparable since the range of both is $[0,1]$. For spots outside the neighborhood (both spatial and pattern) of object spot, we assigned their corresponding contribution to 0, which means they have no contribution to smoothing of the object spot.

In pattern space, to better capture global gene expression patterns, we used PCC distance described in Equation (2) as the distance metric, whose range is $[0,2]$. If the expression of two spots has a negative correlation, the distance based on Equation (2) will become >1. However, smoothing with negative correlation spots is not performed since they are dissimilar. Therefore, we set the contribution to 0 if the pattern distance between the object spot and one of its neighbors is >1. We used an exponential transformation to achieve this goal. For object spot $i$ and its pattern neighbor $j$, pattern contribution ${c}_{ji}^\mathrm{p}$ can be defined as follows:

(3) \begin{equation*} {\displaystyle \begin{array}{c}{c}_{ji}^\mathrm{p}=\left\{\begin{array}{l}\begin{array}{@{}ll}\exp \left(-{\left(\frac{d_{ij}^\mathrm{p}}{\sigma}\right)}^2\right)\ & \kern1em {d}_{ij}^\mathrm{p}< \mathrm{1}\end{array}\\{}\begin{array}{@{}ll}0& \kern7em \mathrm{otherwise}\end{array}\end{array}\right.\kern-6pt.\end{array}} \end{equation*}

The exponential function in Equation (3) limits the range of ${c}_{ji}^\mathrm{p}$ to [0, 1] and ensures that ${c}_{ji}^\mathrm{p}$ decreases as ${d}_{ij}^\mathrm{p}$ increases. $\sigma$ in this equation is a tuning parameter that controls how the pattern contribution decays with pattern distance. When ${d}_{ij}^\mathrm{p}>3\sigma /\sqrt{2}$, ${c}_{ji}^\mathrm{p}$ will quickly decay to 0 [32, 33]. Hence, setting $\sigma$ to $\sqrt{2}/3$ will remove the effect of negative correlation neighbors and we can simplify Equation (3) to

(4) \begin{equation*} {\displaystyle \begin{array}{c}{c}_{ji}^\mathrm{p}= \exp \left( {-4.5{\left({d}_{ij}^\mathrm{p}\right)}^2}\right).\end{array}} \end{equation*}

For spatial neighbors, we used Manhattan distance, an integer >0, as spatial distance to measure their distance to the object spot. Since the inverse proportional function has a similar decay nature as exponential function, we used the inverse of spatial distance as the spatial contribution, i.e.

(5) \begin{equation*} {\displaystyle \begin{array}{c}{c}_{ji}^\mathrm{s}=\frac{\mathrm{1}}{d_{ij}^s}.\end{array}} \end{equation*}

In this case, $i$ is the spot being smoothed and $j$ is the spatial neighbor of spot $i$.

### Padding of missing spots

For ST slides, there are two types of missing values. The first type of missing value is missing genes, which means the spot itself is located in the tissue region, but some specific gene expression is missing. This is the main form of dropout events and also frequently happens in single-cell data, which most of the smoothing methods can handle. The second type is missing spots, i.e. expression of all genes in the spot is 0. These kinds of absent data are unique in ST data and cannot be padded without spatial position information, which often indicates the spot has been removed due to a quality problem. Hence, it is necessary to judge whether the missing spot needs to be padded. As shown in Figure 1**C**, for each blank spot in a slide, SPCS will only pad the ones whose predetermined spatial neighborhood has >50% non-blank spots. This criterion ensures that the boundary of the tissue will not be erroneously expanded. Since there is no expression on missing spots at all, we estimate the expression of missing spots by their spatial neighbors only. Let $i$ become a missing spot, and its expression ${X}_i$ can be estimated by the following:

(6) \begin{equation*} {\displaystyle \begin{array}{c}\mathbf{X}_i=\frac{\sum_{j\in \mathbf{N}_s(i)}{c}_{ji}^\mathrm{s}{\mathbf X}_j}{\sum_{k\in \mathbf{N}_s(i)}{c}_{ki}^\mathrm{s}}.\end{array}} \end{equation*}

In Equation (6), $\mathrm{N}_\mathrm{s}(i)$ is ${\tau}_\mathrm{s}$-order spatial neighborhood of spot $i$, ${c}_{ji}^\mathrm{s}$ and ${c}_{ki}^\mathrm{s}$ are spatial contributions of spot $j$ and $k$ to spot $i$. Spot padding is performed after non-missing spot smoothing. To evaluate the adjusted Rand index (ARI) score for padding spots, we assign ground-truth labels for them. The ground-truth labels of the padded spots are determined by spatial contribution-weighted major voting of their spatial neighbors and manually inspected by a pathology resident.

### Performance evaluation

To evaluate the effectiveness of our proposed SPCS method, we first performed SPCS and other one-factor smoothing methods (SAVER and MAGIC) on PDAC, DLPFC, and simulated ST datasets. SAVER and MAGIC are two representative one-factor smoothing methods using different techniques (i.e. statistical model-based and kNN-based). Then, we partitioned both smoothed and the original unsmoothed real-world slides using the *K*-medoids clustering method [34] and judged how well the clusters were separated after smoothing by internal evaluation of the unsupervised clustering. In addition, we also explored how the parameters in SPCS will influence the smoothing. Next, we performed multiple unsupervised clustering methods, including *K*-medoids, Louvain [35], mclust [36], BayesSpace [28] and spaGCN [37], on all the smoothed and unsmoothed slides to find out how well the clusters match the histopathological labels from the corresponding image as an external evaluation. Simulating data were also used here to detect outliers for each smoothing method. As a gene filter, we only kept genes with <70% zero expressed spots in our analysis. For normalization, we performed logarithmic count per million normalization before smoothing using SPCS, SAVER and MAGIC. The distance metric used during clustering was Pearson correlation distance as described in Equation (2). For each dataset, the number of clusters is predetermined by ground truth, which is provided in Supplementary Tables S1 and S2 available online at https://academic.oup.com/bib. PCA was performed prior to clustering, and the eigenvectors with top 20 eigenvalues were selected to reduce the dimensions of expression matrices and to enhance the clustering results.

### Internal evaluation

For the internal evaluation, silhouette score was used as the evaluation indicator [38]. Silhouette score, a metric whose range is [−1, 1], estimates the average distance between clusters. A greater silhouette score indicates better cluster separations. For an ST spot $i$, let $a(i)$ be the average distance between $i$ and all the other ST spots within the same cluster and $b(i)$ be the smallest average distance between $i$ and all the ST spots with any other clusters. The silhouette coefficient $S(i)$ of ST spot $i$ can be expressed by the following:

(7) \begin{equation*} {\displaystyle \begin{array}{c}\mathrm{S}(i)=\frac{\mathrm{b}(i)\,-\, \mathrm{a}(i)}{\max \left\{\mathrm{a}(i), \mathrm{b}(i)\right\}}.\end{array}} \end{equation*}

The silhouette score of an entire ST slide is the average silhouette coefficient of all spots in it.

In this work, Pearson correlation-based measurement in Equation (2) was used to calculate the dissimilarity between ST spots from the imputed ST data. Traditionally, Euclidean distance is used, but here, we adopted the dissimilarity metric defined in Equation (2) since our clustering method is based on PCC distance. Padded spots are excluded in this analysis. To make the average silhouette coefficient of clustering under different smoothing methods comparable, we used the same distance matrix, which was based on dimensionality-reduced original unsmoothed ST expression. Hence, in our experiments, compared with unsmoothed slides, an increase or lack of change in silhouette score on smoothed slides represents the smoothing method has enhanced the original data distribution, while a decrease indicates the smoothing method has corrupted the original data distribution.

### External evaluation

For the external evaluation, we obtained the histopathological labels of the ST spots. The correspondence between smoothed slides and histopathological labels was evaluated at both clustering and gene expression levels. At the clustering level, the imputed ST data are clustered using multiple clustering algorithms. Technical details of the clustering methods are shown in Supplementary Table S4 available online at https://academic.oup.com/bib. Next, the concordance was evaluated between the clusters from the imputed ST data and the labels from the corresponding histopathological images. Then, the distribution of marker gene expression was compared to the locations of histopathological labels.

The ARI was used to evaluate the similarity between the clustering results from the imputed ST data and the histopathological labels [39]. For clusters from the imputed ST data $\Big\{{\mathbf{I}}_1,{\mathbf{I}}_2,{\mathbf{I}}_3,{\mathbf{I}}_4\Big\}$ and the histopathological categories of ST spots $\Big\{{\mathbf{H}}_1,{\mathbf{H}}_2,{\mathbf{H}}_3,{\mathbf{H}}_4\Big\}$, we denoted ${n}_{ij}$ as the number of ST spots that are in both ${\mathbf{I}}_i$ and ${\mathbf{H}}_j$. The ARI is defined as follows:

(8) \begin{equation*} {\displaystyle \begin{array}{c}\mathrm{ARI}=\frac{\sum_{ij}\left(\begin{array}{c}{n}_{ij}\\{}2\end{array}\right)\,-\,\frac{\sum_i\left(\begin{array}{c}{n}_i\\{}2\end{array}\right){\sum}_j\left(\begin{array}{c}{n}_j\\{}2\end{array}\right)}{\left(\begin{array}{c}n\\{}2\end{array}\right)}}{\frac{\mathrm{1}}{\mathrm{2}}\left[{\sum}_i\left(\begin{array}{c}{n}_i\\{}2\end{array}\right)\,+\, {\sum}_j\left(\begin{array}{c}{n}_j\\{}2\end{array}\right)\right]\,-\,\frac{\sum_i\left(\begin{array}{c}{n}_i\\{}2\end{array}\right){\sum}_j\left(\begin{array}{c}{n}_j\\{}2\end{array}\right)}{\left(\begin{array}{c}n\\{}2\end{array}\right)}},\end{array}} \end{equation*}

where ${n}_i$ is the number of ST spots in ${\mathbf{I}}_i$ and ${n}_j$ is the number of ST spots in ${\mathbf{H}}_j$. A higher ARI value indicates that the imputed ST clusters and the histopathological labels are more similar. In this analysis, padded spots that are unable to be clustered with the ground truth will be treated as an error cluster.

In the marker gene evaluation with the PDAC dataset, two marker genes, *PRSS1* and *TM4SF1*, were used to compare their expression distribution spatially. Both genes are protein-coding genes. *PRSS1* encodes a trypsinogen, which is often highly expressed in normal pancreatic tissues, while *TM4SF1* is a common proto-oncogene and is highly expressed in pancreatic cancer among other malignancies [40, 41]. High expression of *TM4SF1* in the cancerous regions of the PDAC dataset has been detected in previous research [10]. For the DLPFC dataset, three other marker genes, *MOBP*, *PCP4* and *SNAP25*, were used in the analysis. Previous research has reported that these genes can delineate different cortical layers [14]. The expression distribution of these representative genes can stratify the histopathological regions and can be used to measure the accuracy of that partition.

### Biological analysis

To examine how our algorithm aids in informing the biology of an ST sample, we identified differentially expressed genes (DEGs) and performed gene ontology enrichment analysis (GOEA). DEGs were identified in the PDAC slides by comparing two groups of spots with *TM4SF1*-high (neoplastic tissue) and low expression (non-neoplastic tissue). To define the two groups, we first linearly transformed the expression values of *TM4SF1* between [0, 1] by dividing each value by the maximum expression value. The spots on each slide were split into two groups, with one having a transformed expression value <0.7 (*TM4SF1* under-expressed) and the other greater (*TM4SF1* over-expressed). The cutoff 0.7 was chosen as it can reflect the boundary of the cancer region accurately.

A Kruskal–Wallis test was performed between the over- and under-expressed groups, and the *P*-values were adjusted using the Benjamini–Hochberg method to account for the multiple comparisons [42]. Only genes with an adjusted *P*-value < 0.05 were included. In addition, we used logarithmic foldchange (logFC) to determine the up-regulated and down-regulated events for genes. Genes with a logFC > 1 were considered as up-regulated and as down-regulated if the logFC were <−1.

GOEA was performed on the DEGs using ‘g:Profiler’ [43]. The significance of enriched terms was tested by cumulative hypergeometric test, and *P*-values were corrected by g:SCS method [44]. Only terms with an adjusted *P*-value < 0.05 were reported. All data sources offered by g:Profiler were used, including gene ontology (GO) [45], Reactome [46], KEGG [47], WikiPathways [48], TRANSFAC [49], CORUM [50], Human Protein Atlas [51] and the Human Phenotype Ontology [52]. Heatmaps for each sample were then generated to compare the terms found by each smoothing algorithm.

The DLPFC slides were processed slightly differently than the PDAC slides. To find the DEGs, one layer of the cortex was compared with the other six layers. For example, the expression values for all the sports in Layer 1 (L1) were compared to the expression values for the spots in layers L2, L3, L4, L5, L6 and WM (white matter). A Kruskal–Wallis test was performed between one layer of the cortex and all other layers, *P*-values corrected using the Benjamini–Hochberg method and logFC calculated. This resulted in seven sets of comparisons. The same cutoffs of *P*-value < 0.05 and logFC > 1 were used. The significant DEGs were then compared to a set of DLPFC layer marker genes published by Zeng *et al*. [53]. Enrichment analysis was performed using g:Profiler in the same way as the PDAC slides were processed.

## Results

In this study, we applied our two-factor smoothing method SPCS and two state-of-the-art one-factor smoothing methods (MAGIC and SAVER) to smooth ST slides. We first evaluated the computational cost of SPCS and provided the results in Supplementary Table S3. To compare the performance of different smoothing methods, we evaluated the quality of generated clusters after performing unsupervised clustering on both smoothed and unsmoothed expressions. The generated clusters and the distribution of marker gene expression were compared with pre-labeled histopathological partitions to check if the smoothed expression more accurately reflected the pathology features of the corresponding images. In addition, DEGs were identified in different regions and GOEA was performed to reveal the biological meaning from the smoothed data.

### Internal evaluation

**Figure 2:**
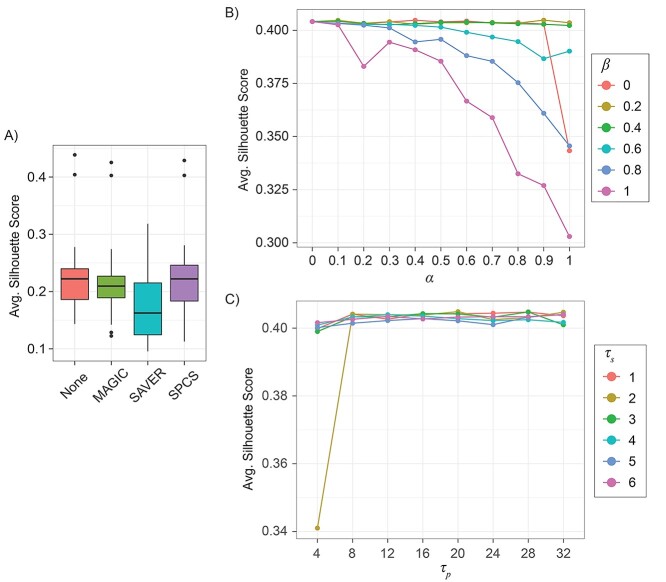
Influence of smoothing on data separability and data distribution. (**A**) Box plot of average silhouette score of 10 PDAC samples and 8 seven-layer DLPFC samples without smoothing and with smoothing by different methods (MAGIC, SAVER and SPCS). (**B**) Influence of parameters $\alpha$ and $\beta$ on average silhouette score for SPCS smoothed PDACA1 slide when ${\tau}_\mathrm{p}=16,{\tau}_\mathrm{s}=2$. (**C**) Influence of parameters ${\tau}_\mathrm{p}$ and ${\tau}_\mathrm{s}$ on average silhouette score for SPCS smoothed PDACA1 slide when $\alpha =0.6,\beta =0.4$.

The silhouette score indicates the average distance between clusters in a slide. Since we used the same unsmoothed distance matrix in the different smoothing methods, the silhouette score reflects how well the smoothing methods kept the original data distribution. For different smoothing methods, a greater silhouette score than an unsmoothed slide usually represents better separability of the smoothed expressions compared to the original data distribution. In contrast, a decreased silhouette score indicates the smoothing method has changed the original data distribution making it less separable. After clustering the spots into the corresponding clusters, we calculated the silhouette score of 10 PDAC slides and 8 DLPFC slides with 7 clusters for each smoothing method, as shown in Figure 2**A** and Supplementary Figure S1 available online at https://academic.oup.com/bib. In most of the slides from these two datasets, SPCS and MAGIC got a similar or even greater silhouette score to the unsmoothed slide, while SAVER got a significantly lower score. In addition, SPCS had the greatest average silhouette score over the other smoothing methods even slightly higher than the unsmoothed slide. This result indicates that kNN-based smoothing generally can keep the characteristics of original data distribution resulting in better data separability.

Figure 2**B** and **C** illustrates how the parameters influenced the data separability of SPCS smoothed PDACA1 slide. For the two ratio parameters $\alpha$ and $\beta$, while $\beta \le 0.4$, silhouette score was not affected much by changing $\alpha$, but the result became more sensitive to $\alpha$ once $\beta$ is large. This result indicates that pattern-based smoothing can help keep the original data distribution. In addition, the results reveal that the data distribution of spatial neighbors is different from the pattern neighbors. For the neighborhood size parameters, the results show that ${\tau}_\mathrm{s}$ and ${\tau}_\mathrm{p}$ have no significant influence on data separability. However, while ${\tau}_\mathrm{s}=2$ and ${\tau}_\mathrm{p}=4$, silhouette score is significantly lower than other parameter combinations, which indicates that neighborhood size parameters also should be well tuned according to the dataset to avoid unexpected results.

### External evaluation

**Figure 3:**
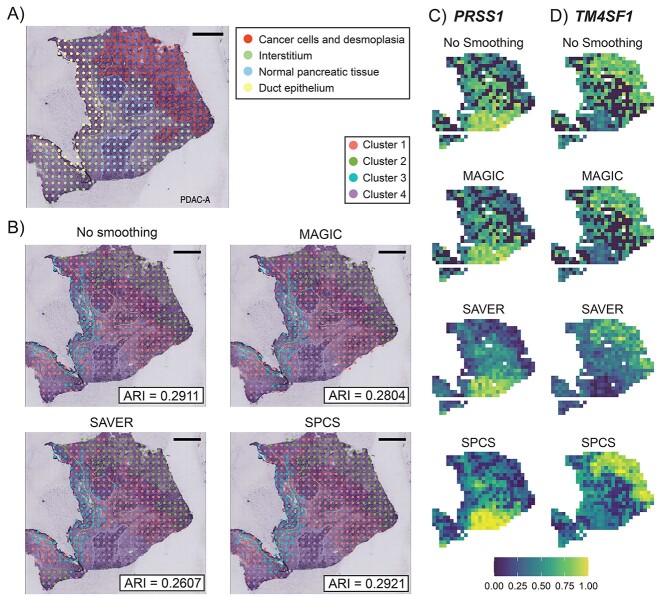
Influence of smoothing on clustering accuracy in PDACA1. (**A**) Original ST slide of PDACA1 and its histopathological partitions. (**B**) Results of *K*-medoids clustering on expression smoothed by different methods. ARI score, which reflects the correlation between clusters and histopathological labels, is marked at bottom-right corner of each figure. Clusters are ordered by their size. Heatmap of smoothed expression of two marker genes, (**C**) *PRSS1* and (**D**) *TM4SF1*, are also shown. For demonstration purposes, expressions of genes are linearly transformed into the range of 0–1 as normalization.

In the external evaluation, different smoothing methods were evaluated based on the consistency of unsupervised clusters with histopathological labels. The result of PDACA1 alone is shown in Figure 3 since the histopathological labels were not available for other PDAC slides. This slide can be well clustered by *K*-medoids clustering even without smoothing. When examining the results in detail, Figure 3**B** shows that most ST spots in the cancer cells and desmoplasia region were separated from other clusters. Most ST spots in the duct epithelium region were also well separated, with slight mixing of the interstitium and the normal pancreatic tissue regions. It is worth mentioning that by including spatial position information, SPCS can pad missing spots, which other one-factor smoothing methods cannot do. Figure 3**C** and **D** shows the influence of different smoothing methods on marker gene expression. Compared with other smoothing methods, SPCS generated a better marker gene contrast between distinctive histopathological areas due to two reasons. First, marker gene expressions showed fewer dropouts and better-reflected expression patterns across different tissue regions with SAVER and SPCS (MAGIC does not impute the missing expressions). Second, and in contrast to SAVER, SPCS imputed the missing values and kept the spatial distribution of non-missing values stable.

**Figure 4:**
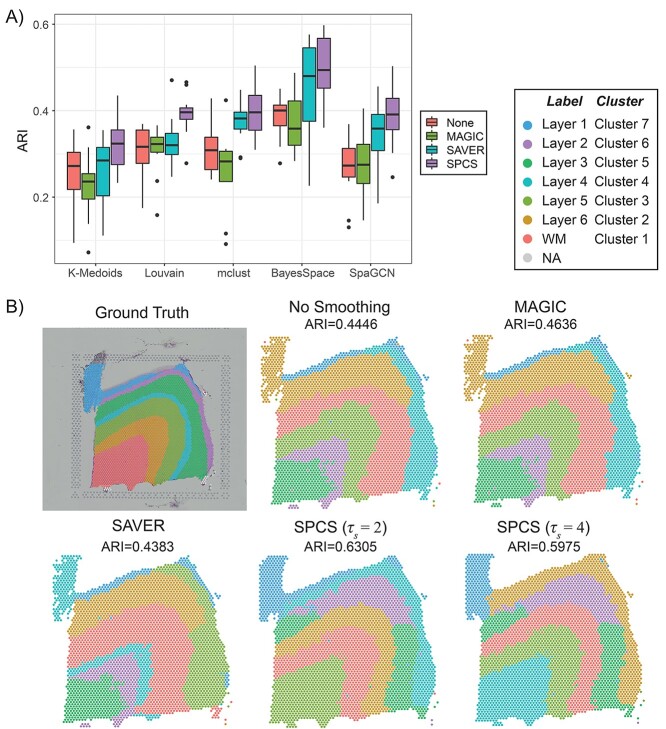
Influence of smoothing on clustering accuracy in DLPFC dataset. (**A**) Box plot of ARI score on various combinations of smoothing (No smoothing, MAGIC, SAVER and SPCS) and clustering methods (*K*-medoids, Louvain, mclust, BayesSpace and SpaGCN) in 12 DLPFC samples. (**B**) Ground-truth label and BayesSpace clustering results of smoothed and unsmoothed sample 151675. Clusters are ordered by their size.

Figure 4 illustrates external evaluation results from 12 slides in DLPFC dataset. Figure 4**A** shows the influence of smoothing methods on different clustering methods. To make the evaluation comprehensive, we choose three commonly used general clustering methods (i.e. *K*-medoids, Louvain and mclust) and two state-of-the-art clustering methods developed specifically for ST slides (i.e. BayesSpace and SpaGCN). The results revealed that smoothing achieved a higher ARI score using various clustering methods and that SPCS outperforms other one-factor smoothing methods for every clustering method. When combining SPCS with Louvain or mclust clustering methods on the DLPFC dataset, we got an ARI score near BayesSpace, indicating that the combination of SPCS with general clustering methods improves the performance of spatial clustering on ST slides. In addition, combining SPCS and BayesSpace got the highest ARI score among all the combinations. Due to the difference in preprocessing steps, the results of SpaGCN clustering were moderately lower than the original publication [37], which indicates that hyperparameter settings of SpaGCN are sensitive to preprocessing steps and we hope to optimize this in the future. Figure 4**B** and Supplementary Figure S2**A**, available online at https://academic.oup.com/bib, illustrate clustering ground-truth and BayesSpace clustering results combined with different smoothing methods on slides 151675 and 151673. Compared with other one-factor smoothing or without smoothing, SPCS can achieve more clear and accurate boundaries between different regions, which contribute to a higher ARI score. In addition, smaller spatial neighborhood (${\tau}_\mathrm{s}$) for SPCS can help to capture long narrow regions in the slide but may cause over clustering in thicker regions with a similar length and width. Similar to the PDAC dataset, SPCS also helps to obtain a better marker gene contrast between distinctive cortical layers in DLPFC dataset as shown in Supplementary Figure S2**B**–**D** available online at https://academic.oup.com/bib.

**Figure 5:**
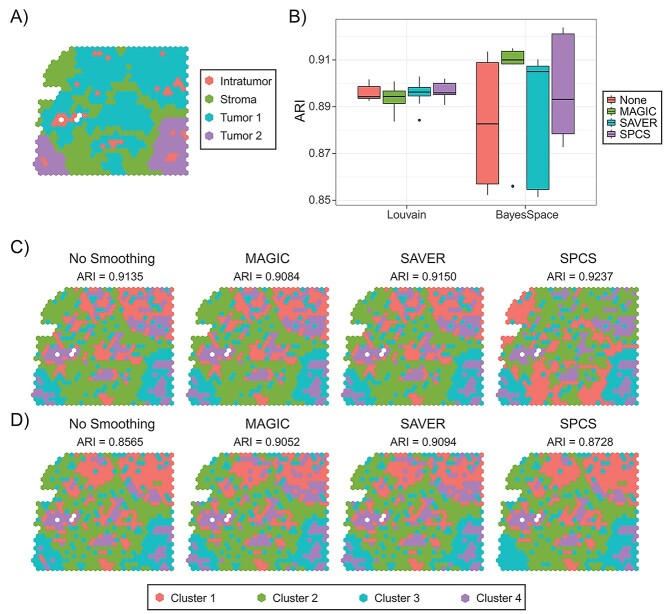
Influence of smoothing on clustering accuracy in HGSOC simulating dataset. (**A**) Ground-truth labels of a simulated ST slide. (**B**) Box plot of ARI score on Louvain and BayesSpace clustered 10 smoothed simulated ST slides. (**C** and **D**) BayesSpace clustering results of two of the simulated ST slides are illustrated. Clusters are ordered by their size.

We also performed simulation analysis to test the clustering accuracy for different smoothing methods. Louvain and BayesSpace, the two best general and ST dedicated clustering methods in the DLPFC experiment, were used in this analysis. The clustering ground truth is shown in Figure 5**A**. For the 10 simulated slides, Figure 5**B** illustrates the distribution of ARI score of each combination of smoothing and clustering method. In general, since the original single-cell dataset was well clustered, every combination of smoothing and clustering methods got high ARI scores in this experiment. With Louvain clustering, there was only a small difference in ARI scores on different slides. For different smoothing methods, SPCS gets a slightly higher average ARI score, which indicates that spatial information helps general clustering methods to cluster ST slides. However, Louvain separates the spots into five clusters instead of four, which means Louvain tends to cluster the spots according to cell types. For the BayesSpace experiments, the ARI scores were lower than Louvain on average and were greater in variance. To better review the higher variance of BayesSpace, we illustrate BayesSpace clustering results of two simulating slides in Figure 5**C** and **D**. Obviously, BayesSpace tends to merge small clusters into nearby larger ones, which leads to oversmoothing in the simulated dataset, and smoothing with SAVER and SPCS will aggravate this problem. Overall, all methods performed well on the simulated data (ARI > 0.85), but it is worth noting that SPCS has a higher potential ARI as evaluated by the 75th percentile. This is surprising considering the other smoothing methods are specifically designed for scRNA-seq data from which these simulated ST slides are generated.

### Biological analysis

**Figure 6:**
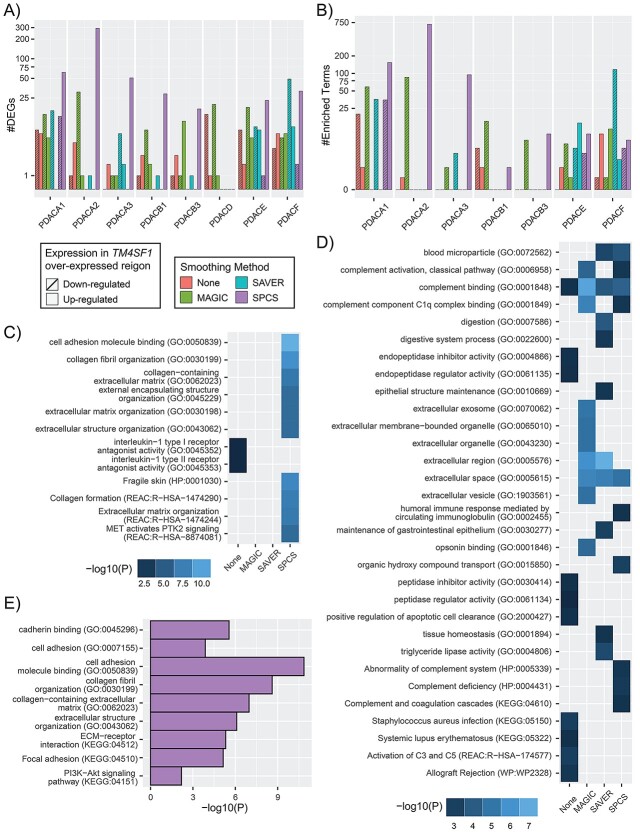
Biological analysis of unsmoothed and different methods (MAGIC, SAVER and SPCS) smoothed PDAC slides. (**A**) Number of DEGs identified in each slide and (**B**) the number of GO terms found from GOEA in each slide shown on a log10 scale. DEGs that are down-regulated in *TM4SF1* over-expressed region and their corresponding GOEA terms are marked as slash-filled texture. The 10 most significantly enriched terms from each smoothing method found in the (**C**) up-regulated DEGs and (**D**) down-regulated DEGs groups in *TM4SF1* over-expressed region for PDACA1 slide are shown, shaded by −log10(*P*-value). (**E**) GO terms enriched in SPCS smoothed slides from up-regulated DEGs that have previously been found in PDAC.

The biological interpretability of the smoothed results was compared between different smoothing methods. Comparing between *TM4SF1* over- and under-expressed regions for the PDAC slides, the significant DEG numbers are displayed in Figure 6**A**. There were no DEGs found in any smoothed or unsmoothed slides of PDACB2 and PDACG because they lacked *TM4SF1*, while a higher number of DEGs were detected by SPCS in six out of the rest eight ST slides. Correspondingly, the number of enriched GO terms was also more from SPCS than the other methods, as shown in Figure 6**B**, which are further examined below.

The top 10 most significant GO terms found in slide PDACA1 for each algorithm are shown in Figure 6**C** for the up-regulated DEGs and in Figure 6**D** for the down-regulated DEGs. More than 10 terms are shown in the heatmap because most GO terms were not shared between smoothing methods. Without smoothing the slides, the up-regulated DEGs identified are related to interleukin-1. Instead, the terms found by SPCS smoothing are related primarily to cell adhesion, extracellular matrix (ECM) organization and MET-activated PTK2 signaling. The GO terms from all smoothed and unsmoothed up-regulated slides can be seen in Supplementary Figure S3 available online at https://academic.oup.com/bib. g:Profiler could not find any enriched terms for slides PDACB2, PDACD and PDACG. Up-regulated enriched terms for all SPCS slides except PDACB1 appear similar to PDACA1. The results for the other methods had a small number of terms which do not seem related to PDAC, except for slide PDACF that contained cell adhesion and ECM terms for the no smoothing and MAGIC.

The enriched GO terms for the down-regulated DEGs (Figure 6**D**) tell a different story. Without smoothing, GO terms related to digestion as well as infection and autoimmune-related pathways and complement cascade components (C3, C5) were identified. GO terms related to digestion were seen with SAVER as well. After performing smoothing, we found many more GO terms related to the ECM, more complement binding, as well as apoptosis regulation, but the infection and autoimmune-related pathways were absent. Applying MAGIC and SPCS also helped to find other complement cascade components such as C1q complex binding. The other slides shown are in Supplementary Figure S3, available online at https://academic.oup.com/bib, and have similar results to the terms described above.

Figure 6**E** contains terms enriched in the SPCS smoothed data from up-regulated DEGs that have previously been reported in PDAC [54–57]. These terms, such as cell adhesion, cadherin binding, PI3K-Akt signaling pathway and focal adhesion, are all significantly enriched in DEGs from SPCS-smoothed data (albeit not among the top 10 enriched terms), but were absent from unsmoothed data, reflecting the enhancement of biological interpretability by performing our novel SPCS smoothing method.

The results of the biological analysis of the DLPFC slides are shown in Supplementary Figure S4 available online at https://academic.oup.com/bib. The significant DEGs are shown in Supplementary Figure S4**A**, available online at https://academic.oup.com/bib, and show a similar number of DEGs for each algorithm. When comparing the DEGs with those reported by Zeng *et al*. [53], a similar number of genes were shared for each smoothing method, as shown in Supplementary Figure S4**B** available online at https://academic.oup.com/bib. These genes include *MOBP* (an oligodendroctye and white matter marker), *PCP4* (associated with L5 and L6) [14, 53] and *GFAP* (associated with astrocytes and many neurological disorders) [58]. In addition, when comparing with other smoothing methods, SPCS helped to discover five DEGs [i.e. Cell Adhesion Molecule 4 (*CADM4*), *ELOVL5*, *AGR2*, *LGALS3BP* and scavenger receptor class B Member 2 (*SCARB2*)], which the authors have not found to be reported in the DLPFC. These genes were found in the white matter layers of the brain. The expression of *CADM4* and *SCARB2* in sample 151673 is shown in Supplementary Figure S4**C** and **D** available online at https://academic.oup.com/bib, respectively.

## Discussion

### Importance of smoothing on ST data

ST data are based on highly multiplexed sequence analysis where barcodes are used to split the sequenced reads into their respective tissue locations. However, this type of sequencing suffers from high noise and dropout events. To keep enough genes to perform biological analysis, we set a relaxed filtering threshold (<70% zero-expressed spots) to filter out non-expressed genes in preprocessing steps. Even with this rather relaxed threshold, <10% of genes in slides of both PDAC and DLPFC datasets were left, which indicated that dropout events are highly frequent in ST datasets. By visualizing the expression distribution of two marker genes, *PRSS1* and *TM4SF1* in Figure 3, the gene-level dropout events can be easily seen, as indicated by the black-colored spots on the slides. *PRSS1* and *TM4SF1* dropout events frequently occurred in interstitium regions of the slide, leading to a failure cluster this area on unsmoothed ST data. In addition, there are also entire spots missing in multiple ST slides, which can negatively influence spatial clustering. By performing smoothing methods, the missing and noisy expression values were controlled to a certain extent so that the partition of regions and downstream analysis are greatly improved. Therefore, smoothing is an important and necessary step for analyzing ST data.

### SPCS improves data quality

Compared with unsmoothed data, smoothing improves data quality. We have demonstrated that various smoothing algorithms increase the separability and partition accuracy of ST spots. Moreover, by performing internal and external evaluations, we confirm that SPCS-smoothed data show better quality as compared with the two existing one-factor smoothing methods, MAGIC and SAVER. In the internal evaluation, from the results shown in Figure 2, SPCS smoothing method produces greater silhouette scores than MAGIC and SAVER, which means the ST data smoothed by SPCS have better separability. In addition, a more similar silhouette score of SPCS smoothed and original unsmoothed data indicates that SPCS can better preserve the original data distribution, which helps to keep accurate biological analysis results while improving spatial clustering accuracy.

Our external evaluation verifies the partition accuracy of smoothed data. One main objective of slide partition is to identify different histopathological regions in the slide. Hence, we measured the degree of overlap between unsupervised clusters on smoothed data and histopathological partitions using ARI. Since the spots in the same histopathological region are usually connected, incorporating spatial knowledge is expected to boost partition accuracy. Moreover, spatial knowledge can also help to detect and pad missing spots. Indeed, as expected, results in Figures 3–5 reveal that SPCS method generates a higher ARI score than existing one-factor methods, which means a more accurate histopathological partition can be acquired by performing the two-factor SPCS method. In addition, it is also clear that SPCS can be used before various clustering methods to improve clustering accuracy. From the marker gene analysis, SPCS method recovered the dropout events and enhanced the expressions of marker genes in the corresponding regions. This evidence proves that SPCS can improve the accuracy of the ST spot partitions.

### SPCS enhances biological interpretability

Our SPCS method identified many more DEGs than the other smoothing methods tested for most of the ST slides. *IL1RN*, *KRT7*, *LAMB3*, *LAMC2*, *NOTCH3 and S100A16* were identified as up-regulated DEGs in the PDACA1 slide after SPCS processing, and each of these genes has been associated with poor survival in PDAC [55, 59]. *IL1RN* and *LAMB3* were also found using unsmoothed data and *LAMB3* was detected with MAGIC smoothed data, but the other genes were uniquely detected by SPCS. A higher number of DEGs associated with GO terms after SPCS processing than unsmoothed, SAVER and MAGIC processing. Specifically, only slides PDACE and PDACF contained up-regulated DEGs and GO terms for SAVER and MAGIC, although PDACD was the only slide that had DEGs and no GO terms for the down-regulated genes. The unsmoothed up-regulated slides PDACA2, PDACB3, PDACD and PDACE and down-regulated slides PDACA3, PDACB3 and PDACD also had DEGs but no GO terms. In contrast, every SPCS smoothed slide that had DEGs also had GO terms. The SPCS data demonstrated a *TM4SF1* expression landscape that matched the original histopathological assignment more accurately than the other smoothing methods.

The GO terms reported in PDACA1 using SPCS related more to pancreatic cancer and to the role of *TM4SF1* than the non-smoothed data and the two one-factor smoothed data. *TM4SF1* has been found to be over-expressed in PDAC and has roles in apoptosis, proliferation and cell migration [40]. Previous work found that collagen 1 (GO:0030199) binds with DDR1, which then interacts with TM4SF1 to activate the focal adhesion kinase (FAK) [40, 60, 61]. This results in the disruption of E-cadherin (GO:0045296), leading to the Wnt signaling pathway and loss of cell–cell adhesion (GO:0050839) [40, 59]. The FAK pathway also increases the expression of N-cadherin (GO:0045296), which results in the migration of cancer cells [40, 59]. TM4SF1 can also activate the AKT pathway (KEGG:04151), leading to anti-apoptotic effects and angiogenesis [40, 62]. All of these previously identified GO terms are important factors for PDAC survival and metastasis. They were also identified using SPCS-smoothed data only, which would have been missed using other smoothing methods or with the unsmoothed ST data.

The terms found through GOEA were more interpretable in the scenario of PDAC and its pathogenesis when applying SPCS smoothing. Many of the SPCS top 10 terms, as shown in Figure 6**C**, and previously reported terms, as shown in Figure 6**E**, are similar to those found in the literature [54–57] and are consistent between slides. Without applying SPCS, some of the top terms found in Figure 6**D** are involved in typical pancreatic activity, such as peptidase regulator activity (GO:0061134), digestion (GO:0007586) and triglyceride lipase activity (GO:0004806), which indicates that important PDAC pathology-related GO terms may be missed when data are not smoothed or not properly smoothed.

Identifying the DEGs in different histological regions and checking their associated GO terms can help evaluate the biological interpretability of a smoothed slide. It is worth noting that the GO (i.e. gene set database) and the enrichment tool can yield different results. The simplest example would be the use of a hypergeometric test to determine enriched gene sets opposed to gene set enrichment analysis, which accounts for the significance of DEGs. The hypergeometric test has a longer history of use and is easily interpretable whereas gene set enrichment analysis is a newer approach. Furthermore, the gene sets themselves differ between databases such as KEGG and GO. There could potentially be more enriched KEGG pathways for one sample and more enriched GO terms for another. For these reasons, we used g:Profiler since it incorporates many gene set databases in the analysis and relies on the well-established hypergeometric testing approach.

Using the cortical layers in the DLPFC slides, we found a similar number of DEGs and shared genes between the smoothing methods. Most of these genes were previously reported. Due to the enhanced contrast, our proposed SPCS method helped to identify five DEGs in the white matter, while the other methods did not. The *CADM4* and *SCARB2*, to the author’s knowledge, have not been identified as white matter markers in the DLPFC. Cell adhesion molecules like *CADM4* play an important role in myelination by oligodendrocytes. Higher expression of *CADM4* leads to many short myelin internodes that disrupt the normal myelination process [63]. *SCARB2* is a lysosomal membrane receptor for the glucocerebrosidase enzyme. It has been associated with Parkinson’s disease and Lewy Body disease. Glucocerebrosidase degenerates sphingolipid, which is important for brain development. There is some evidence that decreases in sphingolipids can lead to demyelination [64, 65]. While the authors do not intend to present *CADM4* and *SCARB2* as marker genes for white matter, we believe that SPCS can be used to help aid with this task. The enrichment analysis results for DLPFC are not shown but are similar between all smoothing methods. This is likely because the number of GO terms found is correlated with the number of DEGs. It is possible that using a gene marker instead of layers to identify DEGs could produce different results. Given the generally clearly defined boundaries of the brain, using layer data to get differential gene expression seems more appropriate.

### Determination of SPCS parameters

There are four parameters in SPCS:${\tau}_\mathrm{s}$, ${\tau}_\mathrm{p}$, $\alpha$ and $\beta$. The parameters ${\tau}_\mathrm{s}$ and ${\tau}_\mathrm{p}$ are designed to adjust the size of spatial neighborhood and pattern neighborhood, respectively. Including more information while performing smoothing is beneficial for a more robust result, and increasing the size of neighborhood is a good way to achieve that goal. Blindly expanding neighborhoods will incorporate some spots that are not similar to the spot being smoothed; therefore, SPCS uses contribution weighting to reduce this effect, which gives the size of both spatial and pattern neighborhoods limited influence on data separability, as shown in Figure 2**C**. In addition, as shown in Figure 4**B**, spatial neighborhood will also influence clustering sensitivity. A smaller spatial neighborhood (${\tau}_\mathrm{s}$) for SPCS can help to capture long narrow regions in slides but may cause over clustering in thicker regions with a similar length and width. Therefore, we recommend a modest selection of these two parameters to balance the trade-off. For most cases, ${\tau}_\mathrm{p}\le 16$ and ${\tau}_\mathrm{s}\le 4$ are recommended.

The parameters, $\alpha$ and $\beta$, are designed to balance the original expression and corrections from spatial and pattern neighbors, which has a significant effect on smoothing quality. Due to the pattern similarity between the object spot and its pattern neighbors, corrections from pattern neighbors tend to enhance the original data distribution features, which is shown by an increased or stabilized on silhouette score. In contrast, corrections from spatial neighbors make the object spot expression consistent with its spatial neighbors. This is beneficial for spatial clustering but may change the original data distribution, leading to a worse silhouette score. To keep the accuracy of spatial clustering and biological analysis simultaneously, it is important to balance the intensity of correction with the underlying expression signatures. Results in Figure 2**B** indicate that the influence of smoothing strength ($\alpha$) on data separability is heavily reliant on the proportion of spatial and pattern correction ($\beta$). Hence, we recommend setting *α* between 0.2 and 0.8 and *β* <0.6 in most cases, and values should be selected carefully according to data distribution. In addition, as indicated in Figure 5, when combined with spatial clustering methods like BayesSpace, smaller $\alpha$ and $\beta$ are recommended to avoid erroneous merging of small clusters.

## Conclusion

In response to expression noise and dropout events in barcoding-based sequencing technologies, smoothing has become an essential data processing step before performing the downstream analysis on ST data. In this paper, we proposed a novel two-factor ST data smoothing method, SPCS, which can take full advantage of both the expression patterns and the spatial patterns contained in ST data. Compared with traditional one-factor smoothing methods, SPCS improved separability, partition accuracy and biological interpretability of ST experiments. SPCS can effectively improve ST data quality for accurate and meaningful downstream analyses. SPCS is broadly applicable to any barcoding-based ST technology.

**Key Points**

- Due to the common issue of noise and dropout events in ST data, smoothing has become a necessary step before downstream analysis on ST data.
- SPCS is a novel kNN-based two-factor smoothing method which can fully utilize both expression pattern and spatial knowledge in ST data.
- Compared with traditional expression pattern knowledge-based one-factor smoothing methods, SPCS can provide better separability, partition accuracy and biological interpretability.

## Supplementary Material

SPCS_supplementary_R1_bbac116

## Data Availability

All of the data used for the analyses in the manuscript are freely available from their original publications.

## Author Biographies

**Yusong Liu** is a PhD student at the College of Intelligent Systems Science and Engineering, Harbin Engineering University. His research focus is on machine learning and network analysis in bioinformatics.

**Dr Tongxin Wang** is a recently graduated PhD student from the Department of Computer Science, Indiana University, who is currently employed as a research scientist at Facebook. His research focus is on deep learning, transfer learning and adversarial networks.

**Ben Duggan** is a medical student at the Indiana University School of Medicine. His research interests include bioinformatics, machine learning and using computing to improve patient care.

**Dr Michael Sharpnack** is a pathology resident at the Department of Pathology at the University of California San Francisco. His research is primarily concerned with novel immunotherapies, antigen presentation and bioinformatics methods development as it applies to lung cancer.

**Dr Kun Huang** is a professor and chair of the Department of Biostatistics and Health Data Science, Indiana University School of Medicine, Indiana University School of Medicine Precision Health Initiative Chair for Genomic Data Science, Director of Data Science and Informatics for the Precision Health Initiative, Associate Director of Data Science for the Indiana University Simon Comprehensive Cancer Center and an Investigator at the Regenstrief Institute. His main research interests are in medical image analysis, multi-omics and machine learning.

**Dr Jie Zhang** is an assistant professor of Medical and Molecular Genetics, who is also a member of the Center for Computational Biology and Bioinformatics. Her research interests are applied translational bioinformatics and systems biology for cancer and neurological disease.

**Dr Xiufen Ye** is a professor of College of Intelligent Systems Science and Engineering, Harbin Engineering University, IEEE Senior Member. Her main research interest includes image processing, pattern recognition and artificial intelligence.

**Dr Travis S. Johnson** is an assistant research professor at the Department of Biostatistics and Health Data Science, Indiana University School of Medicine. His research interests include applied machine learning for use with high-dimensional omic data as it applies to cancer and dementia research.
